# Experiencing challenges when implementing Active Management of Third Stage of Labor (AMTSL): a qualitative study with midwives in Accra, Ghana

**DOI:** 10.1186/1471-2393-14-193

**Published:** 2014-06-05

**Authors:** Stina Mannheimer Schack, Amna Elyas, Gladys Brew, Karen Odberg Pettersson

**Affiliations:** 1 Capio S:t Göran Hospital, Stockholm, Sweden; 2 Region Skåne, Skånehuset i Kristianstad, J A Hedlunds väg, Kristianstad, Sweden; 3 Safe Mother Care Programme, Ghana Health Service, Accra, Ghana; 4 Social Medicine and Global Health, Lund University, Malmö, Sweden

**Keywords:** Active management of the third stage of labor (AMTSL), Post-partum hemorrhage (PPH), Oxytocin, Controlled Cord Traction, Uterine massage, Ghana, Task shifting

## Abstract

**Background:**

Post-partum hemorrhage (PPH) is the major cause of maternal mortality in Ghana and worldwide. Active management of the third stage of labor (AMTSL) is a globally recommended three-step method that in clinical trials has been proven effective in prevention of PPH. The AMTSL guidelines were introduced in 2003, modified in 2006, and has been part of the national guidelines in Ghana since 2008. In 2012, the guidelines were modified a second time. Despite its positive effects on the incidence of PPH, the level of adherence to the guidelines seems to be low in the studied area. This appears to be a problem shared by several countries in the region. An in-depth understanding of midwives’ experiences about AMTSL is important as it can provide a basis for further interventions in order to reach a higher grade of implementation.

**Methods:**

Twelve in-depth interviews were conducted with labor ward midwives who all had previous training in AMTSL. The interviews took place in 2011 at three hospitals in Accra Metropolis and data was analyzed using qualitative latent content analysis.

**Results:**

Our main finding was that the third step of AMTSL, uterine massage, was not implemented, even though the general attitude towards AMTSL was positive. Thus, despite regular training sessions, the midwives did not follow the Ghanaian national guidelines. Some contributing factors to difficulties in providing AMTSL to all women have been pointed out in this study, the most important being insufficiency in staff coverage. This led to a need for delegating certain steps of AMTSL to other health care staff, i.e. task shifting. The fact that the definition of AMTSL has changed several times since the introduction in 2003 might also be an aggravating factor.

**Conclusions:**

The results from this study highlight the need for continuous updates of national guidelines, extended educational interventions and recurrent controls of adherence to guidelines. AMTSL is an important tool in preventing PPH, however, it must be clarified how it should be used in countries with scarce resources. Also, considering the difficulties in implementing already existing guidelines, further modifications must be made with careful consideration.

## Background

Every day, approximately 800 women around the world die from preventable complications related to pregnancy and childbirth [1]. In sub-Saharan Africa, where maternal mortality reaches its peak, the lifetime risk of dying during pregnancy or childbirth is one in thirteen, as compared to an average of one in four hundred in high-income countries [1]. Postpartum hemorrhage (PPH), most commonly caused by uterine atony, is one of the major causes for maternal mortality [2]. In Ghana, where the actual study was conducted, the maternal mortality ratio was 450/100.000 live births between 2005 and 2009 [3]. A survey from 2008 found PPH to account for 24% of all maternal deaths [3], being the single largest cause in Ghana. Primary PPH is defined as a vaginal blood loss of 500 ml or more from the vaginal tract within the first 24 hours of the infant’s birth [4,5]. The most critical period for PPH is the time from the birth of the baby until the expulsion of the placenta, i.e. the third stage of labor. Traditionally, the management of the third stage has been expectant, i.e. delivery of the placenta by gravity, maternal effort and endogenous oxytocin stimulation, not facilitated by uterotonic drugs.

In a joint statement in 2003, the International Federation of Obstetrics and Gynecology (FIGO) and the International Confederation of Midwives (ICM) introduced Active Management of Third Stage of Labor (AMTSL). The original guidelines included administration of uterotonic agents and cord clamping within one minute of delivery of the baby; active separation of the placenta by controlled cord traction (CCT) following signs of placenta separation; and uterine massage (UM) immediately after delivery of the placenta and subsequently every fifteen minutes for two hours [6]. The global consensus regarding the benefits of AMTSL was strongly supported by clinical trials conducted on PPH prevention [7,8], and it was consequently included in the WHO manual *Managing Complications in Pregnancy and Childbirth*[9]. The guidelines were modified in 2006 to include the recommendation of delayed cord clamping (one to three minutes after birth), as this allows a prolonged flow of blood in the cord and thus may improve iron status in the infant. Further, oxytocin was pointed out as the drug of choice in preference to other injectable uterotonics and misoprostol [10]. In 2012, the WHO released new guidelines where the emphasis on CCT was revisited [11]. The new recommendations, based on a study by Gulmezoglu et al., argue that omission of CCT has little effect on the risk for severe hemorrhage [12]. It is therefore considered optional in settings where skilled birth attendants are available but contraindicated in settings where laymen or other health workers assist childbirths. Further, early cord clamping (less than one minute after birth) has since 2012 been generally contraindicated with respect to the benefits that delayed cord clamping may have on the infant’s iron status. Also in 2012, continuous UM was discommended as a component of AMTSL in women who have received prophylactic oxytocin for the following reasons; it may cause maternal discomfort, it requires the presence of a dedicated health professional, and may not lead to a reduction of blood loss. In a recent study by Sheldon et al., the use of UM is not associated with a reduction of PPH incidence but may in fact lead to an increased risk of severe bleeding [13]. However, surveillance of uterine tonus through abdominal palpation is recommended in all women for early identification of postpartum uterine atony. In summary, the use of uterotonics is considered as the main intervention throughout the active management of third stage of labor.

A study in Ghana from 2007 found that AMTSL according to the international ICM/FIGO guidelines was applied in three percent of all deliveries [14]. In 2007, Ghana had not yet embraced the ICM/FIGO standard, but recommended a version of AMTSL that included all of the ICM/FIGO components but also allowed the use of different uterotonics such as ergometrine and misoprostol. When applying the Ghana standard in 2007, the same study indicated a correct implementation in only five percent of all deliveries [14]. Similarly, studies from Uganda [15], Nigeria [16] and Tanzania [17] report improper use of the initial guidelines. Ghana has, since 2008, implemented ICM/FIGO standards for AMTSL in their national guidelines [18]. Nationally, 55 percent of all deliveries take place in health facilities and are assisted by skilled midwives [3], which is a prerequisite for the use of AMTSL. AMTSL is considered a cost-effective intervention [19,20], and maternity services including cost for oxytocin are subsidized for all Ghanaian women since 2008.

In order to understand the factors determining Ghanaian midwives’ level of adherence to the AMTSL guidelines it was deemed pertinent to explore their experiences of using the procedure. Previous studies on AMTSL have focused on quantitative aspects, such as the rate of adherence to AMTSL among surveyed providers. To our knowledge, no qualitative study has been conducted in Ghana or elsewhere in Africa on this subject. By performing this study, we hope to contribute with new insights that might facilitate implementation of AMTSL, not only in Ghana but also in similar settings where this important intervention may not routinely be used.

## Methods

### Study design and setting

In order to obtain in-depth information from each midwife, the study was carried out with a qualitative research design, applying in-depth individual interviews. This method allowed the midwives to share their views freely and provided the researchers with opportunities to probe, i.e. to pursue specific aspects of the research topic [21]. The interviews of this study were conducted in 2011, i.e. before the most recent modification of the international AMTSL guidelines. All interviews were conducted in English. They took place at two district hospitals with three and six delivery beds, respectively, and one regional hospital with 10 delivery beds. The three hospitals were located in Accra Metropolis; the capital of Ghana, which has a total population of approximately 1.7 million [22]. The number of births in 2010 ranged from 2318 at one of the district hospitals to 8133 at the regional hospital.

### Sampling of participants

In order to yield as credible information as possible, the midwives were selected using the purposive method [21] i.e. identifying informants with adequate knowledge of the studied phenomenon. The inclusion criterion for participation in the study was previous training in the use of AMTSL, either though midwifery school, in-service training or a workshop. Midwives with a range of experience of AMTSL i.e. between half a year to three years and representing all shifts were identified in collaboration with the matron in each maternity unit.

#### ***Data collection***

During September 2011, twelve interviews were conducted, ranging from 20 minutes to 1 hour and 30 minutes. An interview guide with the subsequent central topics were developed by the research team: 1) Midwives’ perception of the effectiveness of AMTSL in prevention of PPH during the third stage of labor, 2) Factors influencing midwives’ ability to offer AMTSL to all women in labor and 3) Midwives’ experience of women’s reaction to the AMTSL procedure. The following initial question was used for all interviews; “Would you please explain how you manage the third stage of labor at your working place?” Subsequently, open-ended questions were used in order to encourage the midwife to convey her specific experiences in own words. After satisfactory probing of each topic, each midwife was given the opportunity to add further thoughts on the subject. The last two interviews did not render new valuable information, i.e. we considered that saturation was reached, and no further interviews were therefore conducted [21]. All interviews were tape-recorded and transcribed ad-verbum by AE and SMS.

#### ***Data analysis***

Data was analyzed using the latent content analysis, as described by Graneheim and Lundman (2004) [23]. Latent content analysis is a process where the underlying meaning of the text is interpreted and presented as themes and subthemes reflecting the views and experiences relayed by the midwives during the interviews. Each interview was treated as a text unit and further divided into meaning units ranging from a few words to several paragraphs. These were subsequently condensed into shorter meanings, without removing any important information, and coded. Similar codes were pooled into sub-themes. Care was taken to avoid that codes fell between or fitted into more than one sub-theme, i.e. being mutually exclusive [23]. According to the same principle but with less emphasis on mutual exclusiveness, the sub-themes were merged into themes. Table 1 illustrates the analysis of latent content moving from meaning unit to theme.

**Table 1:** Coding matrix illustrating the analysis of latent content moving from meaning unit to theme

**Meaning Unit**	**Condensed text unit**	**Codes**	**Sub-theme**	**Theme**
Sometimes it becomes difficult if you are alone, you cannot practice AMTSL very well because you may have to attend to an asphyxiated baby.	Practicing AMTSL when baby needs urgent attention is difficult on your own	• Working alone	Being forced to put AMTSL at a lower priority	Facing reality when attempting to implement AMTSL
		• Prioritizing newborn		
		• Neglecting AMTSL		

#### ***Ethical considerations***

Ethical clearance for the study was granted by the Ghana Health Service Ethical Review Committee, and comprised two district hospitals and one regional hospital in Accra Metropolis. All midwives were informed about the purpose of the interview, the voluntary basis of their participation and their right to withdraw from the study at any time. This was presented in a written consent letter, signed by each midwife prior to the interview. Tape-recording of the interviews was performed only after acquiring written permission for this specific purpose. In order to facilitate for the midwives to speak without restrictions and to secure confidentiality, the interviews were held in secluded parts of the labor wards without any colleagues present. Replacing the midwives’ names by numbers ensured de-identification of data.

## Results

**Figure 1:**
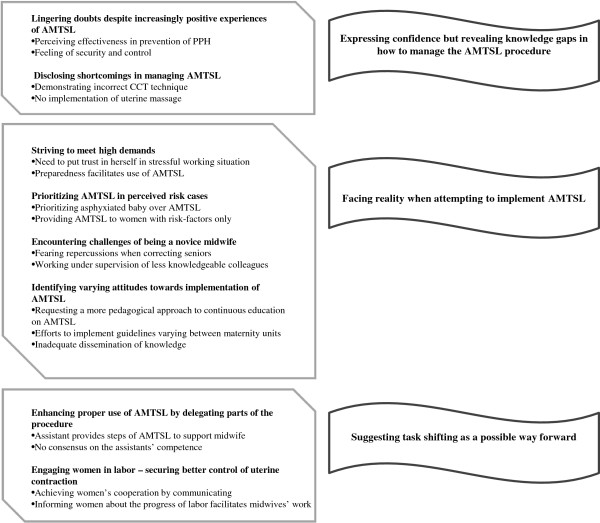
**Themes are presented in banners.** Subthemes (bold) and codes in quadrants.

The findings presented below are based on analysis of the twelve interviews conducted (see Table 2 for characteristics of informants).Three main themes were identified, namely 1) Expressing confidence but revealing knowledge gaps in how to manage the AMTSL procedure, 2) Facing reality when attempting to implement AMTSL and 3) Suggesting task shifting as a possible way forward. These are presented in the text below together with sub-themes and illustrated by quotations from data in italics. After each quotation follows the specific midwife’s number of years of experience at the labor ward. A schematic demonstration of themes, subthemes and codes is presented in Figure 1.

**Table 2:** Characteristics of participating midwives

**Participants**	**Years of experience at labor ward**
1	15
2	2
3	1,5
4	20
5	8
6	4
7	3
8	4
9	1,5
10	21
11	7
12	10

### Expressing confidence but revealing knowledge gaps in how to manage the AMTSL procedure

This theme highlights the midwives’ trust in AMTSL as an effective method in prevention of PPH and their perception that it has become an essential part of everyday work. On the other hand, it brings to attention the fact that knowledge regarding AMTSL to a certain extent is limited, an aspect considered to counteract implementation of the procedure.

### Lingering doubts despite increasingly positive experiences of AMTSL

AMTSL was considered to be more effective than expectant management in preventing PPH during the third stage of labor, as it according to the midwives’ experiences had decreased the incidence of PPH, the number of blood transfusions and the maternal mortality rate at their working place. However, there had been an initial suspicion. Own experience, although initially reluctant, was required to accept and internalize this new course of action. This is demonstrated in the following citation.

*“When they were teaching us* [AMTSL] *I felt, how could you just deliver the placenta when it is not fully separated? I will not come. That’s what I thought. But then I decided to practice it, and when I did, it came. So to me it was like… we were only wasting time previously* [waiting for the placenta]*. So I started to practice it and found that with this method, we did not get the PPH as we used to”*. (M 10, 21 y)

Despite the general perception that application of AMTSL rendered control of the third stage of labor and reduced the incidence of PPH, the view presented below indicate that bleeding was also viewed as an event strongly related to fate and as such not responsive to the administration of oxytocin.

*“I would say that bleeding is an individual event, when the person will bleed she will bleed. If she wouldn’t she wouldn’t bleed. I don’t think the administration of oxytocin is really a contributing factor to the person bleeding or not. It actually helps for the expulsion of the placenta on time. That’s what I know. But for the bleeding aspect, I don’t know”* (M 8, 4 y)

### Disclosing shortcomings in managing AMTSL

When specifically asked to explain the steps of AMTSL as officially practiced in Ghana, the midwives emphasized the importance of administration of oxytocin and controlled cord traction (CCT). However, uterine massage according to the AMTSL guidelines was not referred to by any of the informants.

*“Then after oxytocin you make sure you deliver, because the oxytocin helps to contract and helps in separation of the placenta. But when it separates, if you are not there to deliver it, the cervix closes again”.* (M 5, 8 y)

A fast delivery of the placenta was recognized as the main purpose of AMTSL and the routine for uterine massage varied according to available time and identified risk factors for uterine atony, such as multipara, twin pregnancy and preeclampsia. The midwives often provided uterine massage during the minutes following the delivery of placenta in order to expel clots of blood and reach a primary contracted state of the uterus. In the absence of risk factors, however, and if the uterus was judged adequately contracted, the midwives commonly refrained from further massage.

*“It’s only those where the uterus is flabby and is not contracting, those are the patients we have to be very, very careful with”.* (M 5, 8 y)

### Facing reality when attempting to implement AMTSL

Several factors were pointed out as counteractive to the efforts of implementing the use of AMTSL, two of these being patient overload in relation to staff coverage and non-conducive physical working conditions. Young and inexperienced midwives did not prioritize AMTSL when the work situation was demanding and stressful. Younger midwives also pointed out that communication difficulties with more experienced midwives as well as a resistance among some older colleagues to perform AMTSL were perceived as hindrances. The respondents expressed varying levels of ambition to implement AMTSL, which is evident in the sub-themes presented below.

### Striving to meet high demands

Given the stressful working situation, in combination with uncertain support from midwifery colleagues and/or physicians, the midwives strove to keep “a cool head” and trust themselves despite feeling insecure and at times desperate.

*“You just have to relax and do everything correctly. That’s all. Because there is no help, it’s only you. So just do it”.* (M 8, 5 y)

Relying on divine support to manage in difficult situations appeared to be a common strategy applied by the informants.

*“You panic a bit, but you still have to do what you are supposed to do. You just pray to God to help you; as I’m working, I communicate with God: “please help me, help me do this, help me out”.* (M 7, 3y)

According to the midwives, inadequate space in the delivery ward combined with a high number of women in labor lead to sub-optimal quality of work. A relatively high number of women giving birth on a bench or on the floor was also perceived as an aggravating factor in relation to managing AMTSL properly.

“*About three people are pushing at the same time, some people are pushing on the floor. In that situation there is nothing you can do. So sometimes the active management is not well performed”.* (M 6, 4y)

In addition to the high patient load, the unpredictable progress of labor made it difficult to plan work, especially when working alone. Therefore, the importance of preparation was strongly emphasized by the midwives, particularly regarding the administration of oxytocin, which was due to be given after each delivery. Syringes with oxytocin were consequently prepared in advance in order to avoid any delay.

*“It’s all involving the preparedness, if you have everything there on your trolley. You have your oxytocin syringe, which you can break before the delivery. When you need it you just pick it. It makes things easier when you are prepared for each procedure”.* (M 5, 8 y)

### Prioritizing AMTSL in perceived risk cases

Midwives described situations where they failed to provide oxytocin and CCT. This generally occurred when the midwives were obliged to work on their own and could not call upon assistance. In case of an asphyxiated newborn the midwife always focused on efforts needed to stabilize the baby before returning her attention to the mother. In such a situation, the opportunity to perform AMTSL failed, as indicated below.

*“The baby is delivered and we want to observe vitality within a fraction of a second. You want to prioritize what to do first, because the baby too is very important, so this is where the midwife makes her decision*”. (M 2, 2 y)

Midwives with long experience claimed that they were able to predict if a woman was likely to suffer from PPH or not. The criteria used were previous history of PPH, number of children and amount of bleeding during the second stage. Prioritizing women with such risk factors was considered a useful strategy for dealing with a difficult situation. Besides granting these women oxytocin and CCT, it also implied frequent monitoring of the uterine contraction. Consequently, AMTSL for women without perceived risk factors was not their first priority.

### Encountering challenges of being a novice midwife

Young and newly graduated midwives with limited experience argued that they encountered difficulties in communicating with midwives with longer experience. Although senior midwives stressed the importance of providing a flexible attitude towards their younger colleagues, the latter sensed it was difficult to discuss the AMTSL procedure with their seniors and feared repercussions, as illustrated in the quotation below.

*“We were told to do the counter traction this way* (showing the right maneuver)*, put pressure on the uterus upwards, and then start retracting the placenta. But when I came here, I realized some of my colleagues do it the other way round, they hold the uterus this way* (showing the opposite grip) *and they pull. But they are my seniors so if I say “Don’t do it this way or that way”, they would feel that I was looking down at them”.* (M 9, 1,5 y)

Younger midwives respected the knowledge and experience of their senior colleagues but found it hard to accept that some showed reluctance towards the use of AMTSL. They described how they strove to perform the procedure in a correct manner themselves rather than voicing their opinions. This was partly recognized by older midwives who conceded that implementation of new ideas did not come easy. They agreed that using AMTSL appeared to come more naturally for midwives with recent education and proper training in how to use the procedure, even if they too had undergone in-service training. While acknowledging their own need for more training in the new procedure, they also recognized the problem of being senior supervisors for younger colleagues without possessing the up-to-date knowledge.

*“Some of us pick up things very slowly, especially we the aged ones. So concerning the AMTSL alone, for me, I’ve had my training at least three times. So, it depends on the individual*”. (M 11, 7 y)

### Identifying varying attitudes towards implementation of AMTSL

In general, a positive attitude towards AMTSL was observed, although the engagement displayed in implementation of the practice differed among the hospitals. The midwives expressed interest in more training in AMTSL and recognized the need for evaluation of their current practices, which was considered the responsibility of the in-charges.

*“In fact we have a safe motherhood protocol. I made up my mind, I am bringing one so that we use it to check ourselves. It will be in the ward. I we will be assessing whether we actually are proceeding according to the steps, yes. In time, I think I forgot. But I think I’ll have to do it”.* (M 2, 2 y)

Training sessions on AMTSL were regularly held at different facilities, available for some but not all midwives. After completing a workshop, the participants were expected to disperse skills and theoretical knowledge to their colleagues in the labor ward, through in-service training sessions. As indicated below, feedback from a trained colleague encouraged others to follow suit. Adapting new procedures, however, most likely depend on the active engagement of the midwife in charge of training.

“*We were doing it* [AMTSL]*, but it wasn’t very often, it wasn’t regular…. But after a colleague went to a workshop and came back, she taught us and now everybody is practicing it. But formerly we were not practicing it”.* (M 12, 10 y)

Embracing and implementing change did not only require proper education and follow-up but also personal interest to engage in the process, as indicated below.

“*You can do that* [AMTSL]*, if only you want to. Yes, it’s about the willingness. As a midwife, we are promoting health for the mother and baby, that’s my vision. If you have the passion for it, you can do that”.* (M 5, 8 y)

### Suggesting task shifting as a possible way forward

Midwives often depended on delegating tasks to non-authorized personnel in order to secure a proper use of AMTSL. This was perceived as the key to enhance the use of AMTSL, as one of the characteristics of the midwives’ everyday reality was inadequate staff coverage. Further, efficient communication with the women in labor was pointed out as central for a positive outcome of AMTSL.

### Enhancing proper use of AMTSL by delegating parts of the procedure

Ideally, two midwives should attend every delivery. However, in most deliveries, the midwife attended alone or together with an assistant that might either be a midwifery student or a clinical health assistant with a two-year basic health care education. Depending on her level of knowledge, the assistant was delegated certain steps in the AMTSL procedure normally performed by a skilled midwife i.e. task shifting. The available human resources were consequently used more efficiently in cases of staff shortage, ultimately increasing the use of AMTSL. According to the midwives, the tasks suitable to delegate were administration of oxytocin and monitoring of uterine contraction.

*“I know from observing her* [the assistant] *the things she’s able to do and the things she’s not able to do. If I realize she can administer the oxytocin injection after the delivery of the baby, I can ask her to do that for me”.* (M 11, 7 y)

Whether an assistant should be allowed to perform CCT, however, was controversial, as this procedure was perceived to require midwifery education as well as experience. However, in cases where this task was shifted to a clinical health assistant, the full responsibility of the outcome of this procedure rested on the midwife.

Monitoring of uterine contraction was also viewed as the midwife’s responsibility, but performed according to workload. A strategy embraced by the midwives for maintaining control of the uterine contraction in stressful situations was to teach women how to conduct self-massage of their uterus, as indicated below.

*“If the place is busy, maybe the first one hour, I tell the mother herself to massage the uterus whilst I’m attending to another person. We keep observing and when you are less busy you go and rub it yourself to see if there is any bleeding. If there is no bleeding you go and you continue with what you are doing”.* (M 11, 7 y)

The outcome of such a delegation, however, depended on the quality of the information provided and on the woman’s preparedness to inflict this rather painful treatment on herself in the aftermath of a likewise painful delivery. Though, it was pointed out that this responsibility was never given women who were deemed unable to carry on with the massage.

#### ***Engaging women in labor securing better control of uterine contraction***

According to the midwives, the women in labor perceived AMTSL as an intrusive and painful procedure. Successful management of the procedure therefore relied on the women’s cooperation. However, as long as they were well informed, the midwives reported having no problems convincing them to cooperate, as indicated below.

*“If you tell them that after the baby is born you will give them an injection, they will allow you to do it. But massaging of the uterus, that’s what they find difficult, because of the pain. They try to resist. But we normally tell them that if they don’t do it they might bleed. But you see, there is some resistance”.* (M 12, 10 y)

## Discussion

This study provides an insight into the experiences related to the use of AMTSL in Accra, Ghana. Since the interviews were conducted, the AMTSL guidelines have been modified to focus on the administration of oxytocin, which, according to our findings, most certainly has advantages for the midwives’ working situation. A quite remarkable finding in this study was that guidelines for a procedure recommended both by WHO and the Ghanaian Ministry of Health, designed to prevent a huge threat to women’s health, were not being followed despite regular training sessions. Our main finding was the absence of sustained UM, despite the facts that: 1. it was clearly prescribed as a part of AMTSL in the guidelines in Ghana and 2. all interviewed midwives had participated in AMTSL training. A majority of the midwives even had recent training through workshops and in-service training. These findings correlate with the pattern shown in a quantitative survey from Uganda [18], where UM was also excluded from practice. A study from Nigeria [19] demonstrated a correlation between infrequent use of written learning material and low knowledge of AMTSL among midwives, suggesting a need for more emphasis on recurrent training. Surprisingly, according to the Nigerian study as well as to a study from Tanzania [20], having participated in the pre-service AMTSL training offered by the midwifery program did not correspond to a high level of knowledge and practical application of AMTSL. This might indicate that continuous practical training is crucial once the midwives are deployed and actively work at the labor unit.

Some contributing factors to difficulties in providing the best possible care have been pointed out in this study, the most important being insufficiency in staff coverage. However, seemingly what cannot be accomplished in practice obviously deteriorates in importance, i.e. that UM is difficult to manage and thus not even mentioned in theory. Thus, inadequate adherence to existing guidelines must be regarded as a potential threat to safe medical processes, a problem seemingly shared by other countries in the region [18-20].

The fact that the definition of AMTSL has changed several times since the introduction in 2003 might also influence the midwives’ level of adherence to guidelines. In this context, one may be critical of too recurrent changes in the WHO guidelines, as implementation of radical changes most likely take longer in resource poor setting than in high-income countries. Further research is needed in order to investigate the amount of time needed for new recommendations from WHO to be fully implemented on a national and regional level in low- or middle-income countries. Also, adjustments to current conditions and contexts of each country subjected to a new recommendation ought to be given higher priority. This might especially be required with regards to recommendations designed to prevent medical complications in relation to childbirth, such as AMTSL. Management of complications such as PPH is more difficult in poor settings, as both human and medical resources are scarce. From this perspective, the WHO recommendation from 2012 where focus is taken away from UM, is highly interesting in relation to our finding that midwives in Ghana report lack of time as the greatest obstacle to succeed in providing the AMTSL procedure. By eliminating UM from routine practice, their workload would, in theory, benefit greatly. Further, our finding that CCT might be a time saving procedure is strengthening the recommendation for CCT in environments with trained midwives. However, as midwives in our study indicate that women in labor perceive UM and possibly also oxytocin administration and CCT as painful and intrusive, an interesting topic for further research is the women’s experience of these interventions.

The interviewed midwives recognized task shifting as an efficient way to increase the use of AMTSL. Though, the lack of explicit guidelines for what clinical health assistants were allowed to do was clearly affecting the effectiveness of task shifting at the facilities included in the study. Previous research has not explored the benefits of task shifting when it comes to AMTSL. However, sharing of appropriate tasks in similar contexts is supported by WHO [24]. An interesting question for further research is whether unskilled attendants would be able to administer oxytocin safely.

Novice midwives regarded hierarchical structures as a barrier to dissemination of knowledge regarding AMTSL to senior midwives, who sometimes had not acquired the necessary knowledge themselves through training. As described by the younger midwives, this resulted in adherence to inaccurate practice. In this matter, the more experienced midwives failed in their responsibility to educate and guide the next generation of midwives. Should more recurrent training be implemented for midwives who were not taught AMTSL during education, their knowledge would hopefully improve. Also, clear directives from the hospital management about how to accomplish the third stage of labor would probably increase a correct use of AMTSL and relieve the situation for novice midwives.

One might ask how midwives and others endure to work under conditions where it is physically and psychologically impossible to respond professionally to the overwhelming demands. Relying on faith or fate is apparently one strategy applied by the midwives, and in most cases culturally embedded in the informants’ life-world. Several studies [25,26] focusing on maternal health care in the African context have likewise reported strong adherence to faith and fate.

### Methodological considerations

To enhance credibility [21], the analysis of data was discussed continuously among co-authors. In this context, the importance of the local co-investigator GB was immense, as she had a long experience from the delivery care in Accra. The number of informants (12) in itself is not considered a limitation, as in the qualitative approach we seek to achieve saturation of data, which occurs when no new information is added through further interviews. In order to further increase credibility, focus group discussions where participants would be allowed to critically analyze the results and comment on them, could have been conducted to confirm the researchers’ interpretation of data and clarify the information provided. It should also be noticed that the midwives did not have English as their mother tongue, which might have affected their ability to express their exact meaning during the interviews. Since this study only included midwives who had been subjected to organized AMTSL training, the perceptions about AMTSL among the many midwives who have not been trained were never explored, which is a limitation of this study. This would be an interesting subject for further research since midwives with and without AMTSL training are working side by side in the labor wards of Ghana.

## Conclusions

In order to meet the Millennium Development Goal 5, i.e. reducing maternal mortality by three quarters from 1990 to 2015 [27], the incidence of PPH must decrease. AMTSL is an important tool in preventing PPH, however, it must be clarified how it should be used in countries with scarce resources. Considering the difficulties in implementing already existing guidelines, modifications of guidelines must be made with careful consideration. In light of the new WHO recommendations on AMTSL, the qualitative findings in this study highlight the need for extended educational interventions and recurrent controls of adherence to guidelines. It should be noticed though that this study has clearly identified that training and repeated education sessions alone will not change practice. Identifying and targeting highly influential midwives would probably have a greater effect. In a country where reduction of maternal mortality is of high importance but where the number of skilled midwives is inadequate, one would also benefit from a randomized control trial on the effectiveness of task shifting regarding AMTSL. Moreover, considering the latest WHO guidelines, research on the benefits of implementing a more practical approach to managing the third stage of labor in resource-poor settings would be of great value. If UM could be safely eliminated from routine practice and CCT optional, the situation for the midwives of Ghana would most certainly be considerably relieved.

## Abbreviations

AMTSL: Active Management of the Third Stage of Labor; CCT: Controlled Cord Traction; PPH: Post-partum hemorrhage; POPPHI: Prevention of Postpartum Hemorrhage Initiative; ICM: International Confederation of Midwives; FIGO: The International Federation of Obstetrics and Gynaecology; WHO: World Health Organization.

## Competing interests

The authors declare that they have no competing interests.

## Authors’ contributions

SMS and AE planned the study, carried out data collection and analysis, and wrote the report. GB assisted in ethical application, data collection and analysis. KOP participated in planning the study, data analysis and writing the report. All authors read and approved the final manuscript.

## Pre-publication history

The pre-publication history for this paper can be accessed here:

http://www.biomedcentral.com/1471-2393/14/193/prepub
